# A/B Testing of User Enrollment Forms to Enhance Diversity in the Biomedical Workforce via the National Research Mentoring Network: User-Centered Design Case Study

**DOI:** 10.2196/54532

**Published:** 2024-07-02

**Authors:** Toufeeq Ahmed Syed, Erika L Thompson, Jason Johnson, Zainab Latif, Nan Kennedy, Damaris Javier, Katie Stinson, Jamboor K Vishwanatha

**Affiliations:** 1 University of Texas Health Science Center at Houston, Houston, TX, United States; 2 University of Texas Health Science Center at San Antonio, San Antonio, TX, United States; 3 Northwestern University, Evanston, IL, United States; 4 Vanderbilt University Medical Center, Nashville, TN, United States; 5 University of North Texas Health Science Center at Fort Worth, Fort Worth, TX, United States

**Keywords:** diversity, mentoring, health workforce, underrepresented groups, online platform, user-computer interface, A/B testing, split testing, recommendation algorithm, network of mentors, groups, enrollment

## Abstract

**Background:**

The National Research Mentoring Network (NRMN) is a National Institutes of Health–funded program for diversifying the science, technology, engineering, math, and medicine research workforce through the provision of mentoring, networking, and professional development resources. The NRMN provides mentoring resources to members through its online platform—MyNRMN.

**Objective:**

MyNRMN helps members build a network of mentors. Our goal was to expand enrollment and mentoring connections, especially among those who have been historically underrepresented in biomedical training and the biomedical workforce.

**Methods:**

To improve the ease of enrollment, we implemented the split testing of iterations of our user interface for platform registration. To increase mentoring connections, we developed multiple features that facilitate connecting via different pathways.

**Results:**

Our improved user interface yielded significantly higher rates of completed registrations (*P*<.001). Our analysis showed improvement in completed enrollments that used the version 1 form when compared to those that used the legacy form (odds ratio 1.52, 95% CI 1.30-1.78). The version 2 form, with its simplified, 1-step process and fewer required fields, outperformed the legacy form (odds ratio 2.18, 95% CI 1.90-2.50). By improving the enrollment form, the rate of MyNRMN enrollment completion increased from 57.3% (784/1368) with the legacy form to 74.5% (2016/2706) with the version 2 form. Our newly developed features delivered an increase in connections between members.

**Conclusions:**

Our technical efforts expanded MyNRMN’s membership base and increased connections between members. Other platform development teams can learn from these efforts to increase enrollment among underrepresented groups and foster continuing, successful engagement.

## Introduction

The need for mentoring networks is critical in the biomedical sciences, where the shortage of available mentors contributes to a scarcity of students pursuing biomedical careers. Funded by the National Institutes of Health (NIH) in 2014, the mission of the National Research Mentoring Network (NRMN) is to increase opportunities for mentorship and professional development in the biomedical sciences, especially among underrepresented populations. As part of the NIH-sponsored Diversity Program Consortium, the program works to diversify the biomedical research workforce through programming, training, mentorship, and advocacy efforts [1,2].

The MyNRMN platform [3] enables mentors and mentees nationwide to connect one-on-one or in groups with mentors and peer mentors. As of April 2024, MyNRMN has grown to include over 30,000 members from across the nation, engaging more than 15,700 mentees and 8400 mentors who represent more than 4000 institutions and organizations from all 50 US states and US territories. Within the platform, more than 7800 learners have taken online courses and training and have created more than 500 online collaboration and discussion groups with over 9900 participants. There have been more than 12,000 connections made between mentees and mentors in the network, with strong representation from underrepresented faculty, researchers, and students. To expand the reach of this mentorship platform, the MyNRMN development team continually revises the user interface and platform design and adds new features.

The MyNRMN platform faced the following two challenges: increasing enrollment and expanding network connections. Pivotal aspects of creating an online network are continually increasing the number of people in the network and providing opportunities for members to make connections with others and increase engagement. To increase enrollment, especially the enrollment of diverse users, our team sought to improve the ease and appeal of the enrollment process by using split testing (ie, A/B testing)—a method that is used widely to compare two variations of a form or content [4-9]. For web forms, this testing is accomplished by randomly serving one version of a form to half of the platform visitors and a different version to the other half. A post analysis reveals which variation is the most effective in motivating users to complete a task (eg, enrollment). Additionally, we designed and implemented an array of features to facilitate mentoring connections. This paper details the strategies and underlying technology that supported our efforts. By empowering members from diverse backgrounds to join MyNRMN and connect, our platform expands personal networks and increases career opportunities for those who have been traditionally underrepresented in the biomedical sciences.

In this paper, we describe how we conducted the iterative split testing of three enrollment forms to determine how to increase enrollment in a nationwide networking platform for the biomedical workforce. We also discuss the additional mentoring, networking, and professional development features that we created to further encourage enrollment and increase engagement in the platform.

## Methods

### Overview of MyNRMN

MyNRMN was developed by TAS in 2016 as part of the NRMN. Various forms of individual guided mentoring, free-form mentoring, and group mentoring are offered by the NRMN [1,2]. Prior to the development of MyNRMN, the NRMN online resources consisted of a website with information about the program. Since 2016, the MyNRMN platform has supported many online features and resources for mentors and mentees to connect, seek peer or group mentoring, provide guided online mentoring, and create a network of mentors.

### Enrollment—Attracting Individuals to Join MyNRMN

The first step to finding a mentor was joining MyNRMN. To begin the enrollment process, the users registered by using a third-party service—Auth0 (Okta Inc)—to authenticate their sign-in information via Gmail, LinkedIn, Facebook, or email address and password. This service was especially beneficial when integrating the social sign-ins, particularly while modifying the required registration fields throughout this study.

**Figure 1.**
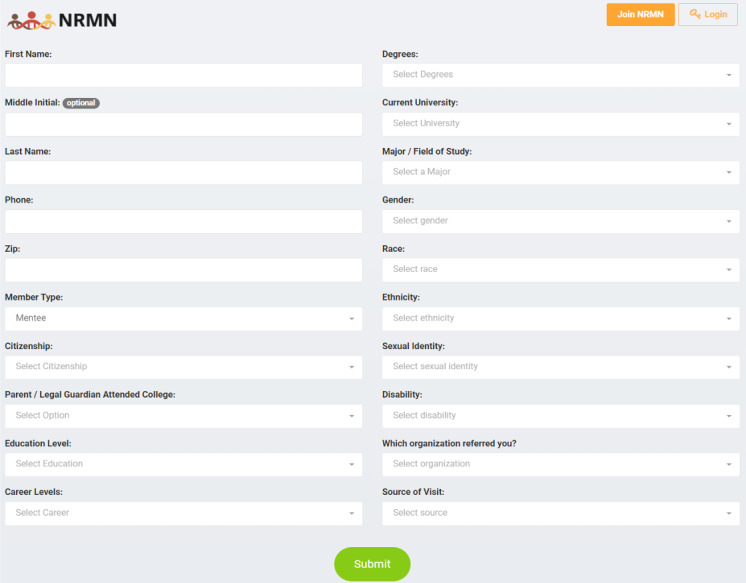
Legacy enrollment form. NRMN: National Research Mentoring Network.

To finish the process, users completed an online enrollment form that included demographic information. An enrollment form was already used at the time Vanderbilt University Medical Center inherited the MyNRMN registration process during the Phase II U24 award period. This “legacy” form, which was designed primarily for data collection, included 19 fields arranged in 2 columns, with all fields required to complete enrollment (Figure 1). The lengthy slate of questions contained potentially sensitive fields, such as fields for ethnicity, disability, and gender. We observed that many users dropped out of the enrollment process after creating an Auth0 account and before completing the form. We became concerned that having sensitive questions early in the process hindered our efforts to recruit diverse members. An additional fear was losing potential members by not offering alternative log-in methods, particularly logging in via social media, which younger users may prefer.

**Figure 2.**
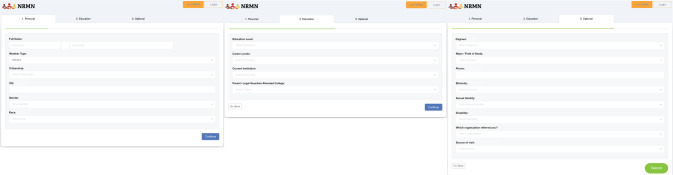
Version 1 enrollment form. NRMN: National Research Mentoring Network.

In hopes of increasing enrollment completion, we created the version 1 form and tested it against the original legacy form. The version 1 form asked the same questions in an improved user interface. In contrast to the legacy form, the version 1 form used a wizard format with 3 tabs. The first 2 tabs presented 11 required fields, while the third tab contained optional fields (Figure 2). When implementing the multipage format, we anticipated reduced perceptions of the form being burdensome.

**Figure 3.**
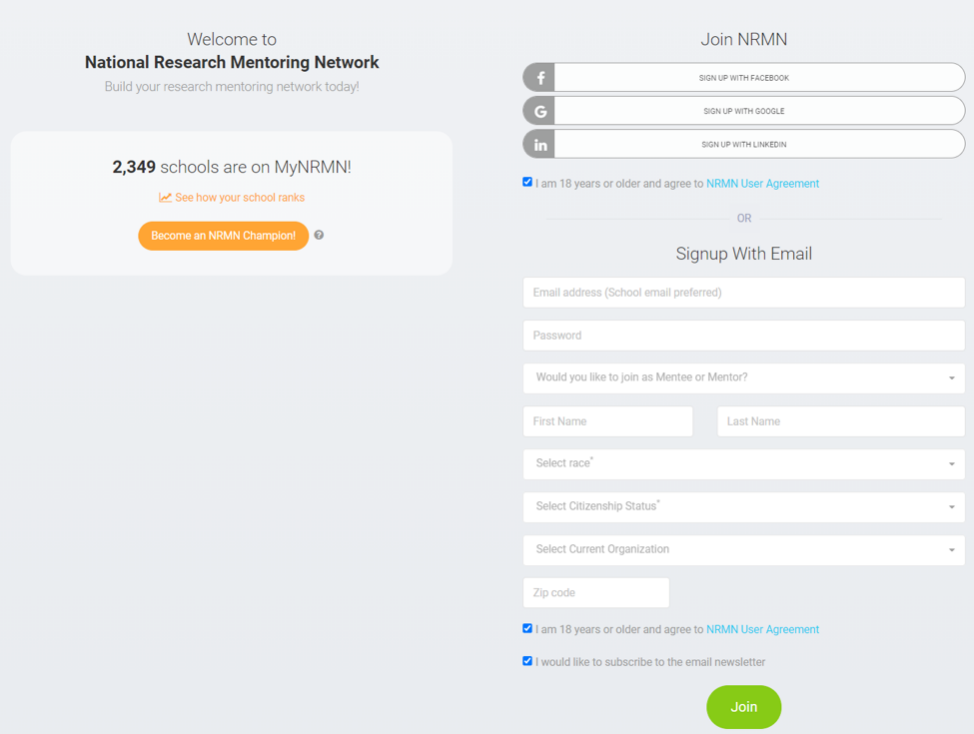
Version 2 enrollment form. NRMN: National Research Mentoring Network.

From July 1, 2019, to August 26, 2020, we used split testing to evaluate the different registration forms to optimize enrollment. To conduct split testing, we programmed the system to randomly redirect each user, after Auth0 sign-in, to 1 of 2 enrollment forms—the legacy form or the version 1 form. Despite seeing some improvement in enrollment among users who were provided with the version 1 form, the improvement was not as significant as anticipated. This motivated us to continue revising the form to develop the version 2 form (Figure 3). The version 2 form allowed users to create an Auth0 account and enroll synchronously. Sensitive questions were removed from the first steps in the version 2 form, and members were prompted, but not required, to complete these questions after enrollment. We observed that removing the requirement to complete the sensitive fields prior to enrollment significantly increased user enrollment. The NRMN’s goal is to increase diversity in the biomedical workforce. To measure the impact of our mentoring platform, we collect demographic data during the user enrollment process. Beginning with the version 2 form, the only demographic information that has been collected during enrollment is race. Since August 26, 2020, all new users have been redirected to the version 2 form, which replaced the legacy and version 1 forms.

### Connections—Finding a Mentor and Building a Personal Network of Mentors

The MyNRMN platform’s goal is to help members increase connections to build and expand their networks. The larger the network, the greater the opportunity to engage with members who are diverse with respect to gender, racial and ethnic background, and other demographics. To facilitate finding and acquiring new connections on MyNRMN, we developed the following new features and modes:

1. Member search engine: Members use the powerful search engine within the *Find a Mentor* feature to search for mentors or peer mentors. Keywords are used to search by name, institution, location, or areas of interest. Additionally, the keyword search is applied to curricula vitae, résumés, and publications that are synced within the platform via natural language processing–based indexing and retrieval.
2. Recommendation engine: Through the use of Neo4j (Neo4j Inc)—a robust graph database technology—the platform’s recommendation algorithms suggest new connections to members. Fresh recommendations appear when a member accesses their personal dashboard.
3. Profile: A member’s profile page contains detailed information, including research interests, institutions, locations, and publications, and excludes any profile questions that may be deemed “sensitive.” A mentee can view the profile of any mentor and request to connect.

### Data Analysis

We estimated descriptive statistics for variables in SAS 9.4 (SAS Institute Inc). To examine the effect of form type on form completion, odds ratios (ORs) and 95% CIs were estimated for odds of completing enrollment by form type and within demographic subgroups. Subgroup analyses were conducted for demographic subgroups with sufficient data available (eg, White, African American, and Asian race subgroups) and on the basis of which fields were required for enrollment completion. After implementing the version 2 form, some demographic fields were converted to optional fields, which resulted in some users leaving these fields blank, hence the incomplete (“Missing from Total”) data in our data set.

### Ethical Considerations

This study was approved by the NRMN’s institutional review board (reference number: 2015‐0720). All user data were protected under and provided by the North Texas Regional Institutional Review Board and stored securely.

## Results

### Growth in Enrollment

Prior to August 26, 2020, a user joining MyNRMN was required to complete the following two steps: (1) create an account with Auth0 and (2) fill in all required fields in the enrollment form. Our analysis showed improvement in completed enrollments that used the version 1 form when compared to those that used the legacy form (OR 1.52, 95% CI 1.30-1.78). The version 2 form, with its simplified, 1-step process and fewer required fields, outperformed the legacy form (OR 2.18, 95% CI 1.90-2.50).

**Figure 4.**
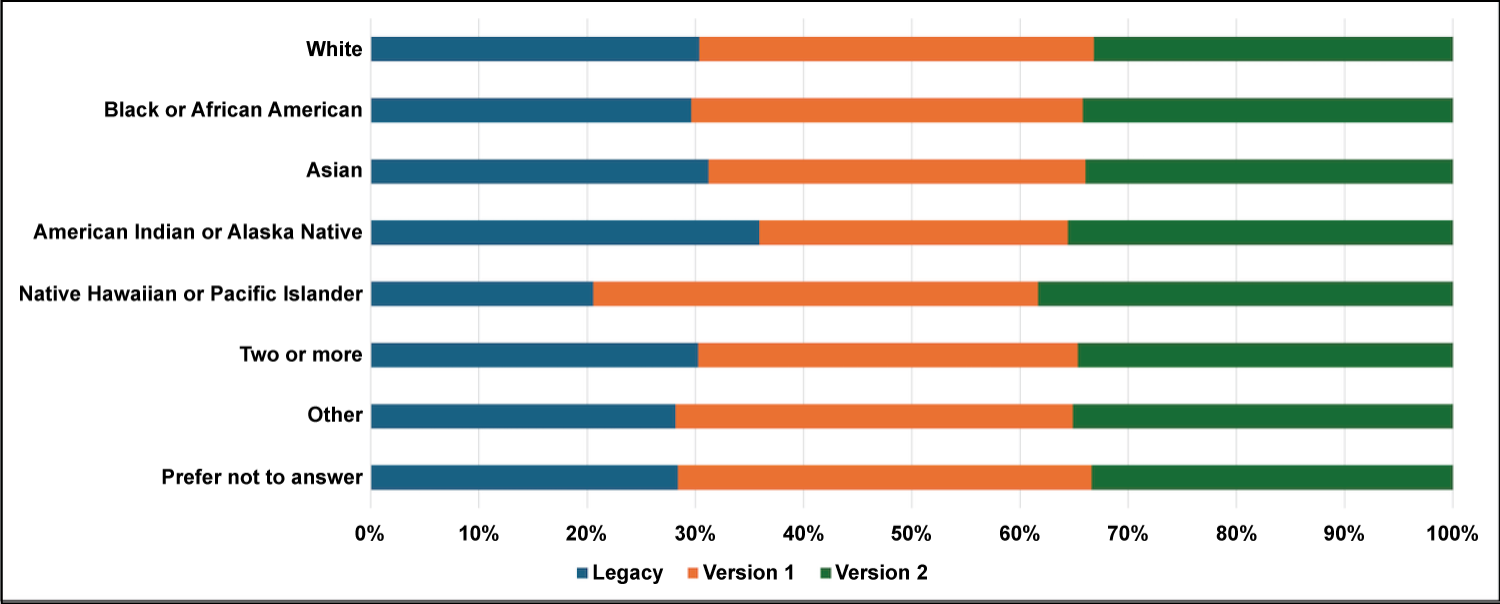
Proportions of users who completed the enrollment form by form type and race.

Table 1 describes the proportions of users who completed enrollment by form type and demographic characteristics. Figure 4 displays the users who completed enrollment by form type and self-identified race. In the version 1 form, the field for ethnicity was optional. In the version 2 form, the fields for ethnicity, gender, and education were optional.

**Table 1.** Proportions of users who completed enrollment by form type.

		Legacy form (N=1368)	Version 1 form (N=1396)	Version 2 form (N=2706)
Overall completion, n (%^a^)		784 (57.3)	938 (67.2)	2016 (74.5)
**Race^b^**				
	White, n (%)	361 (80)	420 (96.1)	958 (87.4)
	Black or African American, n (%)	165 (78.6)	234 (95.9)	395 (90.8)
	Asian, n (%)	128 (84.2)	125 (94)	338 (91.6)
	American Indian or Alaska Native, n (%)	15 (88.2)	7 (70)	21 (87.5)
	Native Hawaiian or Pacific Islander, n (%)	2 (50)	3 (100)	14 (93.3)
	Two or more, n (%)	15 (83.3)	28 (96.6)	42 (95.5)
	Other, n (%)	30 (71.4)	54 (93.1)	114 (89.1)
	Prefer not to answer, n (%)	68 (73.1)	66 (98.5)	135 (86)
	“Missing from Total,” n	381	415	438
**Ethnicity^b^**				
	Non-Hispanic, n (%)	590 (80.4)	632 (95.9)	358 (90)
	Hispanic, n (%)	92 (78)	102 (82.9)	53 (85.5)
	Other, n (%)	31 (77.5)	30 (88.2)	17 (81)
	Prefer not to report, n (%)	71 (80.7)	62 (95.4)	27 (90)
	“Missing from Total,” n	388	515^c^	2195^c^
**Role^b^**				
	Mentee, n (%)	504 (76.4)	602 (86.4)	1313 (97.8)
	Mentor, n (%)	280 (75.7)	335 (85.2)	704 (98.6)
	“Missing from Total,” n	338	306	649
**Log-in method^b^, n (%)**				
	Username-password	556 (58.5)	650 (70.4)	1302 (83.9)
	Google	156 (54.7)	170 (58)	446 (56.7)
	Facebook	20 (48.8)	30 (57.7)	20 (45.5)
	LinkedIn	52 (57.1)	87 (68.5)	249 (77.1)
**Gender^b^**				
	Male, n (%)	236 (81.1)	260 (92.9)	168 (89.8)
	Female, n (%)	526 (77.7)	663 (92.3)	419 (89.2)
	Other, n (%)	2 (100)	3 (100)	7 (100)
	Prefer not to report, n (%)	20 (74.1)	11 (84.6)	2 (100)
	“Missing from Total,” n	371	382	2040^c^
**Education^b^**				
	Undergraduate, n (%)	171 (84.2)	202 (87.5)	85 (84.2)
	Nondegree postbaccalaureate, n (%)	22 (88)	17 (89.5)	8 (88.9)
	Graduate, n (%)	153 (73.9)	175 (90.7)	95 (88)
	Postdoc, n (%)	114 (81.4)	127 (89.4)	89 (89.9)
	Other, n (%)	324 (76.1)	416 (86.1)	228 (91.2)
	“Missing from Total,” n	367	328	2139^c^

a Percentages were calculated by using the N values in the corresponding row headings as the denominator.

b The percentages in these rows were calculated by using the total number of users who self-identified as each subgroup for each form type as the denominator.

c Became an optional field; this “Missing from Total” value includes users who completed enrollment and users who did not complete enrollment.

We performed subgroup analyses to determine if enrollment completion differed within demographic subgroups by form type (Table 2). We compared the legacy and version 1 forms for gender, race, education, and log-in method subgroups, since these were required fields. The version 1 form performed better than the legacy form for male users, female users, White users, Black or African American users, Asian users, graduate students, nonstudents, and persons who used the username-password method. We compared the version 2 and legacy forms for race and log-in method subgroups, since these were required fields. The version 2 form performed better than the legacy form when users used the username-password method (version 1: OR 1.69, 95% CI 1.39-2.04; version 2: OR 3.70, 95% CI 3.07-4.46) or LinkedIn (version 1: OR 1.63, 95% CI 0.93-2.85; version 2: OR 2.52, 95% CI 1.55-4.12). The version 2 form also performed better than the legacy form in terms of facilitating enrollment completion among White, Black or African American, and Asian users.

**Table 2.** Odds ratios (ORs) and 95% CIs for enrollment completion within demographic subgroups.

		Version 1 form vs legacy form (reference), OR (95% CI)	Version 2 form vs legacy form (reference)^a^, OR (95% CI)
**Gender**			
	Male	3.03 (1.76-5.21)^b^	—^c^
	Female	3.46 (2.49-4.81)^b^	—
**Race**			
	White	6.16 (3.60-10.54)^b^	1.73 (1.29-2.32)^b^
	Black or African American	6.38 (3.13-13.03)^b^	2.69 (1.70-4.28)^b^
	Asian	2.93 (1.27-6.77)^b^	2.04 (1.16-3.62)^b^
**Education**			
	Undergraduate	1.30 (0.76-2.24)	—
	Graduate	3.43 (1.93-6.10)^b^	—
	Postdoc	1.93 (0.97-3.83)	—
	Other	1.96 (1.39-2.75)^b^	—
**Log-in method**			
	Username-password	1.69 (1.39-2.04)^b^	3.70 (3.07-4.46)^b^
	Google	1.14 (0.82-1.59)	1.08 (0.82-1.42)
	Facebook	1.43 (0.63-3.26)	0.88 (0.37-2.05)
	LinkedIn	1.63 (0.93-2.85)	2.52 (1.55-4.12)^b^

a Gender and education were not required fields in the version 2 form.

b Indicates statistical significance (*P*<.05).

c Not applicable.

### Growth in Connections

**Figure 5.**
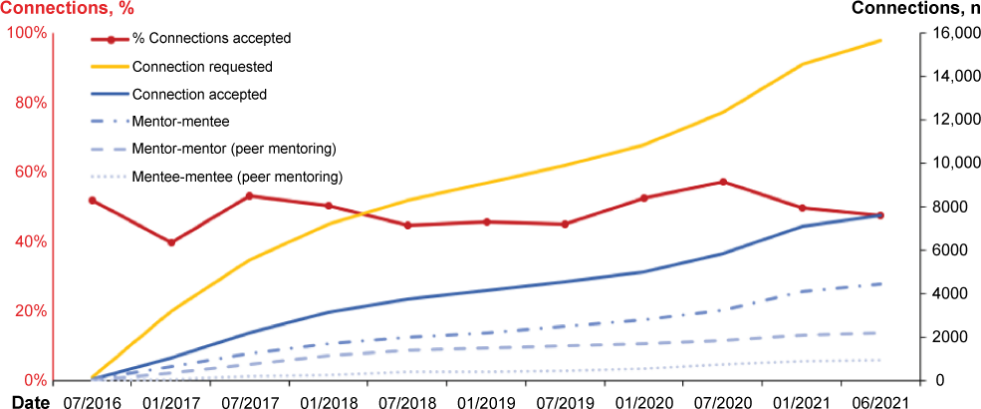
Cumulative mentoring connections.

After implementing the version 2 form, the amount of mentoring requests grew for all types of mentor-mentee connections, including peer-to-peer connections (Figure 5). However, the percentage of accepted connection requests has remained relatively consistent. Of the total requests to connect, roughly half have been accepted. Although very few requests have been actively declined, a large percentage remain pending.

Networking connections originated via several pathways (Table 3). Both mentors and mentees made extensive use of the *Recommendations* feature that suggests connections on the user dashboard. Via the *Search* and *Profile* connection pathways, mentees initiated more connections than mentors.

**Table 3.** Origins of connection requests.

Pathway	Mentees		Mentors	
	Number of connection requests	Requests accepted, n (%)	Number of connection requests	Requests accepted, n (%)
*Recommendations* feature	601	231 (38.4)	724	285 (39.4)
*Search* connection pathway	577	277 (48)	331	171 (51.7)
*Profile* connection pathway	633	320 (50.6)	148	74 (50)

## Discussion

### Principal Findings

Providing access to mentors and meeting the need for mentoring are essential to grow and enhance the biomedical workforce, and the value of mentorship in accessing the connections needed to advance professionally has been long established [10,11]. Research shows that the probability of individual success increases when multiple mentors with differing backgrounds and levels of expertise are tapped for support [12]. This *network of mentors* facilitates growth that is tailored to an individual’s goals by providing the multiple perspectives needed for professional and personal growth [13-15].

Our results show that our enrollment forms work similarly well for female and male users, and the version 1 form performed better (enrollment rate of male users: 260/280, 92.9%; enrollment rate of female users: 663/718, 92.3%) for both groups than the legacy form (enrollment rate of male users: 236/291, 81.1%; enrollment rate of female users: 526/677, 77.7%). This is an important consideration in decreasing traditional gender disparities in the biomedical sciences—a situation that the NIH has recognized by designating female individuals as underrepresented. Although the version 1 form was established as superior to the legacy form in terms of enrollment completion, it did not perform as anticipated. We discussed the shortcomings of the existing forms with MyNRMN stakeholders, including the NRMN-Resource Center, the Product Council (which included representative NRMN members), and the NIH project scientist. After collaborative discussions, we simplified the enrollment process in the version 2 form. We discovered that the design of the user interface did not negatively affect enrollment, which was instead affected by the length and sensitive nature of the information required. After reducing the number of required questions and moving sensitive questions to an optional postenrollment form, our rate of registration completion increased significantly (*P*<.001; Table 1). Moreover, improved registration completion was observed for racial subgroups that used the version 2 form when compared to those that used the legacy form.

In fostering mentoring connections, we found that most mentors (724/1203, 60.2%) extensively used more passive features, such as the *Recommendations*. In contrast, mentees were more likely to proactively use the *Search* feature or the *Profile* feature to build connections when compared to mentors. Both of these features use the personalized recommendation algorithm and natural language processing when providing results. The recommendation algorithm pulls information from a user’s profile, as well as from their network connections within the platform. To promote the further expansion of personal networks, we developed the following five new features: (1) the *Groups* feature (members can join discussion and collaboration groups to connect with others who shared common interests); (2) the *Ask Me A Question* feature (members can ask questions prior to requesting a connection and then initiate the connection request); (3) the *Invites* feature (members can invite peers, colleagues, or friends to join MyNRMN and connect); (4) the *My Cohort* feature (institutions and organizations can bring their members to the platform to connect within the cohort and throughout MyNRMN); and (5) the *Administrative Match* feature (mentees can request a system administrator to initiate a mentoring request to a mentor). Because these features are relatively new, we did not include them in our results. Favorable initial data, however, demonstrate the value of continually adding novel features. Providing the online platform to facilitate mentoring connections, in conjunction with providing additional professional development resources and multiple, easy-to-use enrollment options, has aided the growth of MyNRMN, which now has 30,000 users (as of April 2024). Developing new strategies to promote networking connections may further support MyNRMN platform engagement for additional resources.

We were successful in increasing enrollment on our platform and understanding users’ preferences for the enrollment form. However, we identified a limitation in our A/B testing process, and we believe that a longer testing period and more iterations could provide us with more data for enhancing the user enrollment process. In our future work, we will aim to study how we can encourage participation from underrepresented groups, such as Native Hawaiian or Pacific Islander individuals or people who do not identify within the gender binary.

### Future Directions

The MyNRMN platform’s goal is to ensure that mentoring connection requests are seen and are acted upon. Requests to connect are too frequently languishing, especially those made through the *Recommendations* feature; these may appear less personally targeted than those made through other features. Therefore, we are examining how to strengthen the relevancy of our recommendation algorithm by using machine learning and our graph database for more tailored recommendations. To nudge those receiving a connection request, the platform will remind members immediately upon logging in and will also send email reminders. To ensure that the enrollment and connection emails are received, we will continually monitor spam filter rules and comply with changes and updates that are implemented by email services. In addition, pending requests for connections will be displayed prominently on the dashboard or as a pop-up.

Our goal is to quickly increase each new member’s network from the initial node of 1 to an ever-widening web of connections. We plan to begin including embedded recommendations in a monthly email. In addition, when a connection is accepted, the confirmation email will include additional recommendations.

### Conclusion

We built a platform for online mentoring to meet the needs of our members and to add value by increasing their connections. By conducting the A/B testing of enrollment forms, we were able to identify and overcome a barrier to enrollment and thus provide mentoring, networking, and professional development resources to a broader audience, which in turn promotes the diversification of the biomedical workforce. We continue to evaluate our paradigm and improve our engagement. Many of our ideas are generalizable to those building other membership networks. These entities can learn from our experience that creating multiple pathways, such as by providing social media options for account creation, achieves better results than a single track and that removing sensitive questions, such as those about gender and sexual identity, can attract a more diverse membership. For MyNRMN, our key value proposition is increasing network connections, which we achieve through the technology solutions described herein. By making the enrollment process less onerous and sensitive, ensuring that new members feel instantly welcome, and constantly developing and implementing novel engagement features, MyNRMN fosters an environment that engages an increasingly diverse population of mentors and mentees.

## Abbreviations

NIH: National Institutes of Health
NRMN: National Research Mentoring Network
OR: odds ratio
